# Annotations

**Published:** 1900-01-06

**Authors:** 


### ANNOTATIONS.

### Cremation.

DR. FARQUHARSON, M.P., has recently been lecturing upon cremation, the advantages of which he urges with much embellishment and many more or less apt illustrations. Perhaps universal cremation will come in time; in the meanwhile could we not improve our system of death certification and registration? As Dr. Farquharson says, when a person dies who has not been attended by any medical practitioner there is no provision made for any examination being made before burial, and registration is accepted without question unless some obviously suspicious circumstances become known to some one who may demand a coroner's inquiry. Statistics, he says, show that in 1890 nearly 16,000 dead were smuggled into their graves without any certificate, and that in about 26,000 cases the death certificates given were reported by the Registrar-General as so inadequate as not to be capable of classification. This is surely a sufficiently serious condition of affairs, and to our minds it stands entirely separate and distinct from the question of cremation. Truly, if ever cremation becomes the ordinary and recognised mode of disposing of the dead it will have to be accompanied by a very careful system of registration of the cause of death, and in many cases this will have to be preceded by a complete examination of the body. But this is just what is wanted whether we get cremation or not. Cremation without proper safeguards will never be acceptable, but these safeguards are themselves useful and desirable for their own sake. A complete reform of the whole system of death certification and registration is then the first step to aim at. When this has once been attained the main objections to cremation will have been removed, and the whole question will be reduced to one of cost and sentiment.

### Endowment Funds.

THE ragged mendicant shivering at the street corner and drawing alms from a benevolent public by sheer force of misery is typical of a mode of appeal for charity which is far too commonly made use of by hospital authorities. It seems to be thought that any admission that there is money in hand will dry up the charity of the giver, and therefore every little device that can be thought of is employed to keep the balance on the wrong side. One very favourite plan is that of keeping a capital or endowment account into which legacies and such like large sums are at once diverted, with the effect that while the secretary is issuing heartrending appeals for aid and showing by plain figures that the bills cannot be paid and that wards must be closed unless help is given, the treasurer is turning over the pages of the "Investor's Manual," wondering where the dickens he is to invest his accumulations. A cottage hospital was recently opened at Skipton, a building which had been erected to commemorate the Diamond Jubilee of her Majesty the Queen. The ceremony was performed by Lady Frederick Cavendish, and the proceedings excited considerable interest in the district. For our present purpose, however, we are most concerned about the balance-sheet submitted to the subscribers, which ran as follows: To donations and collections to building fund (paid), £5,191; ditto (unpaid), £395; interest, £69. By transfer to endowment fund, £2,000; payments on account of land and buildings, £2,743; professional fees, £281; other expenses, £100; estimated further liabilities on account of building and furnishing, £2,112; leaving a balance against the hospital of £1,581. It will be seen that the result of this little manipulation leaves the hospital in the lowest depths. Like the shivering mendicant, it can appeal strongly, by aid of its deficiencies, for the assistance of kind-hearted humanity. The "balance against the hospital" is spoken of as being £1,581. But £2,000 have been transferred to an "endowment fund," so that after all the hospital is £419 to the good! But according to the creed accepted by so many hospital managers, it would never do to go a-begging with such a pocketful; so, exactly after the manner of the wealthy crossing-sweeper, the money is banked, and the beggar appears in as miserable a guise as if he had not got a penny. But these tricks of trade are not altogether to our liking, and we are glad to know that in the present day they seldom or never pay.

### Celibacy Among Working People.

LORD ROWTON, in describing the arrangements in the latest of the Rowton-houses recently erected in London, claimed that the building supplied a perfect hygienic home, with all the conveniences of a first-class working men's club, and he pointed out that in a Rowton-house a man could get everything necessary for his comfort for 1s. 3d. a day. This is what seems to us the serious part of the business, and its gravity is by no means lessened by the statement made by Sir R. Farrant that he had recently received an offer from an American gentleman to provide £50,000, free of interest, for the purpose of establishing houses of the same kind for women, that this offer had been entertained, and that a design was in contemplation. Not a word do we say against the philanthropic work undertaken by Lord Rowton. Nay, more, so long as what he does remains but a drop in the bucket of London's great wants in regard to house accommodation we are ready to assert that it will do great good. But it is idle to shut one's eyes to the fact that the great demonstration given by the Rowton-houses of the possibilities of co-operative living, if it should lead to a large extension of the system, as it surely will, must tend greatly to aggravate the hard lot of those who from their being married cannot avail themselves of these advantages. Nothing is more certain than that wages are fixed, not by what the employers choose to give, but by what the workmen are willing to accept, and this is and always will be fixed by the cost of living. When, then, we see that a single man can enjoy the comforts of a "perfect hygienic home" and a "first-class working men's club," and have 9d. a day of spending money left after providing himself with food, and all this for a wage of 14s. a week, we cannot but see that the tendency of the whole scheme is to pull down wages. This does not matter so long as it is on a small scale, but now that we find philanthropists, public companies, the County Council, and even gentlemen in America combining to enlarge and to extend all over the metropolis a scheme which, while acting as a heavy premium upon celibacy, at the same time tends to drag down the wages on which married men even now can hardly live, we must admit that, although with the best intentions, the establishment of these great celibate establishments tends to undermine family life and all that that connotes.

